# Detection of traumatic stress in the presence of traumatic experiences: the role of resilience factors in foster care children five years or younger

**DOI:** 10.1186/s13033-023-00610-w

**Published:** 2023-11-15

**Authors:** Kimberly I. Tumlin, Amanda Crowley, Brian Turner, Elizabeth Riley, John Lyons

**Affiliations:** 1 Center for Innovation in Population Health, College of Public Health, Department of Athletic Training and Clinical Nutrition, College of Health Sciences, University of Kentucky, 720 Sports Center Drive, Lexington, KY 40536 USA; 2 College of Public Health, University of Kentucky, Lexington, KY 40536 USA; 3 Foundation Direct, Lexington, KY 40536 USA; 4 Center for Innovation in Population Health, Department of Health Management & Policy, University of Kentucky, Lexington, KY 40536 USA

**Keywords:** Traumatic stress, Detection, Young children, Foster care, Resilience

## Abstract

**Background:**

Children less than five years of age comprised approximately 30% in 2020 of foster care entries in the United States, and they are consistently the largest foster care entry group. Very young children can respond differently to the same adverse life events. Detection of complex interpersonal traumas is core to providing appropriate interventions and prevention of reoccurring negative outcomes in these children.

**Methods:**

Children who (1) were identified as having experienced complex interpersonal trauma, but (2) who did not have traumatic stress symptoms were identified using Child and Adolescent Needs and Strengths data in a large midwestern state from 2010 to 2021. A logistic model was fit to determine the effect of cumulative traumatic exposures (e.g., adverse childhood experiences such that increased events were hypothesized to predict an increased likelihood of symptomatic detection. We conducted a latent class analysis to understand the relationship between traumatic experiences, asset-based factors, and the detection of traumatic stress in children aged five years and under who had exposure to traumatic events but did not have detectable traumatic stress symptoms.

**Results:**

We detected three classes within this population of very young children, who were described as “resilient” (demonstrating asset-based resilience when faced with traumatic experiences), “missed” (those who exhibit behavioral and mental health types like those with detected traumatic stress symptoms but who were not detected as such), and “unfolding”. Very young children do demonstrate asset-based resilience when faced with traumatic experiences.

**Conclusions:**

Detection of traumatic stress may be more difficult in young children. It is important to assess both traumatic stress and strengths to ensure that children who are resilient after exposure to traumatic experiences (i.e., do not demonstrate traumatic stress symptoms) are not referred to unnecessary interventions. Additional educational approaches are needed to help caseworkers identify symptoms of traumatic stress that mirror symptoms of other behavioral and emotional challenges. Precision medicine approaches are required to best match the interventions to specific needs of young children. Recognition of resilience in very young children is critical for designing systems that customize approaches of trauma-informed care.

## Background

There is a substantial body of research documenting the adverse impact of traumatic events on both short- and long-term wellbeing and functioning [1–4]. The body of research following the seminal work of Felitti et al. identifying a set of ten adverse childhood experiences (ACEs) provided compelling evidence of the negative life outcomes associated with experiencing traumatic events [5]. ACEs research has spawned several measures of that inventory these traumatic experiences [6–8].

More than 33% of all children have experienced at least one traumatic event before the age of sixteen [9]. Seminal work by Cook et al. [10] highlights that some children experience complex interpersonal trauma (CIT); a combination of two or more traumatic events that involved other individuals in their life. This is different from traumatic experiences that are environmental in nature, such as an accident or natural disaster [9, 11]. Examples of CIT include abuse, neglect, and physical violence which often result in children developing symptoms of traumatic stress, including re-experiencing symptoms, intrusive thoughts/memories, avoidance behavior, and changes in cognitions/beliefs [12]. Children who experience CIT, relative to other adverse events, were more susceptible to challenges with their physical, psychiatric, and neurodevelopment as they age [13]. Self-regulation and ability to create interpersonal connections were also negatively affected in children with CIT [10].

Perhaps no group is at greater jeopardy to experience CIT were children served in foster care systems [14]. Studies have estimated that 90% of children in these systems have experienced trauma in their lifetime [15], and these traumatic experiences come in various forms: physical or emotional, real, perceived, direct or indirect, and in single or multiple occasions [16]. Traumatic experiences such as child abuse and neglect resulting in interpersonal trauma can lead to the removal of a child from abusive environments and placement into the child welfare system. Children may have additional traumatic experiences while in the system, resulting in feelings of separation and loss after removal from their home or family environment [14]. Although experiences through traumatic events and subsequent traumatic stress is common in children, CIT often goes undetected and unreported [14, 17], and can manifest as developmental delays and behavioral health challenges [15].

One of the Healthy People 2030 high-priority public health issues is to increase delivery of evidence-based treatments to children and adolescents with symptoms of trauma [18]. However, not everyone who experiences adverse life events develops symptoms of traumatic stress. Some children with history of CIT have instead demonstrated high levels of resilience in the face of adversity [19–21]. Factors affecting resilience development including parental support and caregiver attachment are associated with decreased impact of ACEs [22]. Promotion of these attachments has been an emphasis of building resilience in this the face of prior adversity [23, 24]. In this context resilience is more than ability to cope, rather it is a combination of characteristics which permits adaptation to trauma exposures [19, 21]. Essential aspects of resilience are complex and multi-dimensional, including interpersonal skills, self-regulation, and positive adaptations [21]. The lack of symptomatic development can make standard approaches in trauma-informed systems challenging, because different children respond differently to the same adverse life events [25]. Under these circumstances, using experience-based assessments such as those evolved from the 10-item ACEs [6–8], as opposed to clinical- or symptom-based assessments to guide intervention might lead to inappropriate assignment of trauma interventions to children who demonstrate resilience after experiencing a traumatic event [22]. Furthermore, understanding underlying outcomes which contribute the resilience as a set of asset-based qualities is imperative for children in foster care.

Latent class analysis (LCA) is a form of mixture modeling which is widely used in data analysis to determine the population heterogeneity [26]. Modeling using Child and Adolescent Needs and Strengths (CANS) data has been previously completed for evaluation of strengths in children with mental disorders [27], and to characterize longitudinal behavioral health changes over time while in services [28]. Sex, history of foster care, and mental health needs were associated with different trauma patterns when using the CANS trauma domain in LCA [29]. The cumulative number of ACEs, and impact of combination of these ACEs on child health outcomes using the National Survey of Children’s Health was reported to have seven profiles [30]. For instance, mental illness and poverty ACEs contribute to higher likelihood of poor health—even higher risk than those children who experience more than 7 ACEs [30]. As demonstrated by prior research, the LCA approach permits distinction of unobserved groups who share patterns of predictors or outcomes as those with known trauma exposures. The value of the LCA in understanding trauma exposures and subsequent health outcomes is valued as a person-centered approach [27–31]. A gap in current research is understanding how and why, despite cumulative trauma exposures, some children do not exhibit traumatic stress symptoms. Thus, we aimed to examine this gap by understanding the underlying heterogeneity of populations of children by using LCA to move the field towards care tailored for individuals.

The present study used data collected in a child welfare system to understand the relationship between exposure to traumatic events and the development of traumatic stress symptoms in young children. Specifically, we sought to identify and better characterize young children who were exposed to CIT experiences, and yet were not detected as having symptoms of traumatic stress. Given past findings, we hypothesized these ‘undetected’ children include those who are clearly ‘missed’ and those who are demonstrating resilience in the face of exposure to these traumatic experiences.

## Methods

### Data source

We used data from children aged 0 to 18 years who were taken into state custody and placed in a foster care setting over a period of seven years within a large Midwestern state’s child welfare system. A CANS assessment [32] was completed by the caseworker as an output of the initial comprehensive, team-based assessment process at entry into custody. All data were routinely collected by participating jurisdictions/agencies during normal course of business. No direct contact was made between study personnel and families associated with these data. As such, a waiver of consent was granted according to the University of Kentucky ethics review (#55938).

### Sample

Our sample for this project was from 88,086 observations in the primary dataset representing 15,883 unique children ages 0 to 5 years with documented CIT exposure. CIT exposure was determined as an assessment of two or more traumatic exposures (ACEs; captured by actionable ratings on corresponding CANS items) which included sexual abuse, physical abuse, neglect, emotional abuse, and/or witness to family violence. A total of 8269 (52%) were identified as males. Most children were classified as white (9060, 57%) followed by Black/African American (5127, 32%), Native American/Alaskan (1104, 7%), and Asian (195, 1%). A total of 10% (1615) were identified as Hispanic.

### Instrument

The CANS is an evidenced-based assessment of needs and strengths of children and adolescents and includes an assessment of whether the child experienced any of the thirteen ACEs [33–35]. We used the definition of CIT experiences developed for the CANS by Kisiel, et al. [36]. Specifically, this definition requires the presence of a history of at least two of the following ACEs: sexual abuse, physical abuse, emotional abuse, neglect, and witness to family violence. The CANS version used in this study was adapted from a version developed in collaboration with the National Childhood Traumatic Stress Network [36] and is consistent with the version used in prior research. Embedded within this version of the CANS are 13 common ACEs [32].

The CANS is a communimetric tool designed from communication theory [37, 38]. As such, each individual item is reliable on its own and the ratings of items translate into action levels.

For the *needs* items, the following action levels are used:

0 is no evidence, no need for action.
1 watchful waiting/prevention/further assessment.
2 action (functioning is impaired).
3 immediate or intensive action (dangerous or disabling).

For the *strengths* items, the following action levels are used:

0 centerpiece strength/focus of a plan.
1 useful strengths.
2 identified strength (but must be developed to be useful).
3 no strength is identified.

For purposes of clarity “actionable items” were defined as those CANS need items rated a ‘2’ or ‘3’ indicating an area for targeted clinical intervention or strength identification and building. Individual items that score ‘0’ or ‘1’ were considered “non-actionable.” Strengths were seen as present if rated a ‘0’ or ‘1’.

### Statistical analysis

**Fig. 1:**
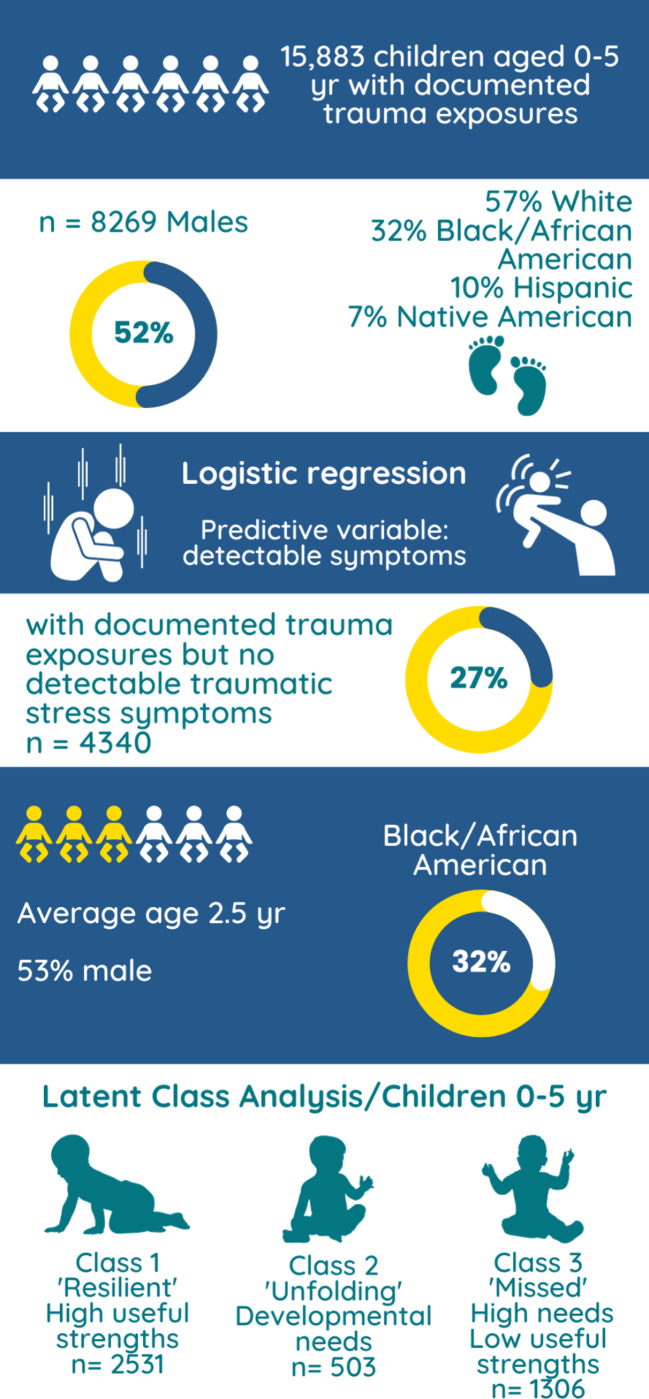
Graphical description of sampling and statistical analyses used to evaluate trauma exposure and expression of traumatic stress symptom data from the Child and Adolescent Needs and Strengths assessment in a large midwestern state from 2011 to 2021

All analyses were conducted using R statistical software (version 3.6.3). Logistic regression was used to predict the relationship between the number of ACEs experienced by the children and the presence of any symptoms of traumatic stress. The total number of ACEs captured by actionable ratings on corresponding CANS items was regressed on the presence of observed traumatic stress symptoms. The presence of traumatic stress symptoms was defined as the child having an actionable rating on one or more CANS items reflecting traumatic stress. Data were coded into binary scores for analysis based on whether they were considered “actionable” (non-actionable = 0, actionable = 1). Cumulative traumas (ACEs) were used to predict expression of traumatic symptoms. The predictive variable was a rating of ‘2’ or ‘3’ on the CANS item of “adjustment to trauma”, meaning that symptoms of traumatic stress were detected and required clinical care. From this logistic regression we developed a subset of children who fell into the category of not having detectable traumatic symptoms. In an exploratory analysis, we then used Latent Class Analysis (LCA) to predict profiles of these children from the original sample (see Fig. 1). The probabilities of each actionable item were determined. We expected between three and six patterns, which are differentially associated with clinical need and strength.

## Results

**Fig. 2:**
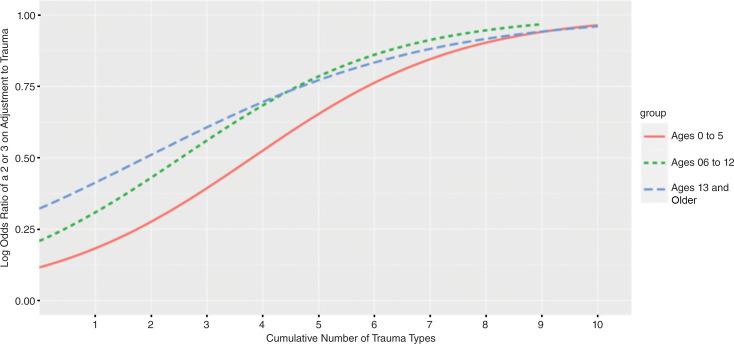
Logistic regression of number of trauma types predicting actionable^a^ adjustment to trauma scores from the Child and Adolescent Needs and Strengths (CANS) comprehensive assessment from a large mid-western state in the United States (2011–2021). ^a^Actionable refers to rating of ‘2 – action’ or ‘3 –immediate/intensive action’ in the Child and Adolescent Needs and Strengths assessment, meaning that some level of action should be taken

Initially we compared this data sample with two other age groups in a logistic regression model (see Fig. 2). The regression model was fit to ascertain the effect of cumulative number of ACEs experienced on the likelihood that the children had detected symptoms of traumatic stress, while controlling for age group (e.g., 0 to 5 years; 6 to 12 years; 13 and older). The logistic model was statistically significant, *X*^*2*^ (3, *n* = 16,683) = 2640, p < 0.001 and explained 19.8% (Nagelkerke R2) of the variance in the data. More cumulative number of ACEs experienced increased the likelihood that symptoms of traumatic stress were detected (OR = 1.53, 95% CI [1.50, 1.57]). Children aged 6 to 12 years old (OR = 2.06, 95% CI [1.90, 2.25]) and children ages 13 and older (OR = 2.19, 95% CI [2.01, 2.78]) were both over twice as likely to have detected traumatic stress symptoms compared to children aged five years and younger.

Results of this logistic regression indicated that detection of traumatic stress symptoms based on ACEs was different among age groups. Of the overall population of children aged five and under, 11,543 (73%) were identified as having CIT exposure with detected traumatic stress, and 4340 (27%) did not have traumatic stress symptoms in the presence of documented traumas. Compared to the children and adolescents above 6 years of age, those with CIT exposure and without detected traumatic stress symptoms were an average age of 13 years, 35% male, and 31% Black/African American. Therefore, we elected to further study this 27% of the population to determine profiles of need and strengths in very young children who have history of trauma exposures but did not have detectable traumatic stress symptoms.

We extracted data on children under the age of five who had experienced CIT but had no actionable traumatic stress symptoms (*n* = 4340). Demographics were summarized in Table 1. The LCA was conducted using all items of the CANS assessment, excluding those items that corresponded to ACEs, to identify classes of asset-based and mental/behavioral health need factors. A total actionable need score was derived by summing any need which was scored a “2” or a “3” for each class. Class 1 had the lowest total actionable need score (score of 5) with Class 3 being moderate (score of 14) and Class 2 having the highest summed actionable need (score of 18). We first fit a one class model and subsequently added classes until we determined a model with the best fit. We followed the recommendation of Nyland et al. [26] that the Bayesian Information Criterion (BIC) served as our statistical criteria, where the lowest BIC results in the best model fit. The BIC reached a minimum at a three-class solution (BIC:3 = 532,187, df = 6825, parameters n = 560). Class 1 comprised 58% of the sample with an average age of 2.5 years and the majority were white females. Classes 2 and 3 had higher percentage of males, with Class 2 having nearly 37% Black/African American children. The average age for class 2 was 2.0 years and this class was the smallest percentage of the total sample (n = 503, 12%, see Table 1).

**Table 1:** Demographics of three classes from Latent Class Analysis of data for children five years and younger from the Child and Adolescent Needs and Strengths assessment in a large midwestern state from 2011–2021

Demographic	Class 1	Class 2	Class 3
Sample n (%)	2531 (58)	503 (12)	1306 (30)
Average Age (years)	2.5	2.0	3.1
Male (%)	49	55	54
Black/African American (%)	31.6	36.4	31.7
White (%)	56.34	55.1	58.2
Other Race (%)	12.1	8.6	10.1

Probability of actionable needs for all CANS factors were summarized by each class determined using LCA (see Table 2). Children in Class 1 (n = 2531) demonstrated the lowest percent of needs among the three classes. Within all domains the only actionable need reported was “family nuclear life functioning”. Children in Class 2 (n = 503) represented a group who had a greater proportion of developmental and medical functioning needs which were actionable. In addition, this group had more actionable “impairment in functioning” than either of the other two classes. Children in Class 3 (n = 1306) had the highest prevalence of needs in the life functioning domain. Like Class 2, these children demonstrated developmental challenges, but also had actionable “attachment behaviors”, “communication functioning”, and “family extended life” needs. Additional needs in Class 3 were observed in “organizational complexity”, “physical functioning”, and “social functioning”.

**Table 2:** Probabilities of actionable^a^ needs by class membership of data for children five years and younger from the Child and Adolescent Needs and Strengths assessment in a large midwestern state from 2011–2021

	Class 1	Class 2	Class 3
	n = 2531 (58%)	n = 503 (12%)	n = 1306 (30%)
	Resilient	Unfolding	Missed
**Behavioral and Emotional Needs**			
Anxiety	0.04	0.09	0.25
Attachment	0.11	0.17	**0.34**
Atypical Behavior	0.00	0.02	0.05
Depression/Withdrawn	0.01	0.02	0.06
Failure to Thrive	0.02	0.15	0.03
Impulsive/Hyperactive	0.04	0.07	0.23
Oppositional	0.02	0.05	0.15
**Child and Family Cultural Factors**			
Cultural Identity	0.00	0.02	0.03
Culture Stress	0.01	0.03	0.05
Expression of Distress	0.04	0.10	0.13
Help Seeking Congruence	0.04	0.08	0.13
Knowledge Congruence	0.03	0.08	0.11
Language	0.02	0.02	0.04
Traditions and Rituals	0.00	0.02	0.02
**Life Functioning**			
Autism Spectrum	0.00	0.02	0.02
Chronicity	0.01	0.26	0.01
Cognitive	0.02	0.17	0.14
Communication	0.13	0.27	**0.37**
Daily Functioning	0.02	0.17	0.24
Dental	0.10	0.09	0.20
Developmental	0.10	**0.34**	**0.33**
Diagnostic Complexity	0.00	0.21	0.00
Eating	0.01	0.14	0.07
Elimination	0.00	0.06	0.05
Emotional Control	0.01	0.10	0.16
Emotional Response	0.00	0.17	0.00
Family Extended Life Functioning	0.13	0.21	**0.32**
Family Nuclear Life Functioning	**0.32**	**0.31**	**0.43**
Impairment in Functioning	0.01	**0.39**	0.00
Intensity of Treatment	0.00	0.14	0.00
Life Threat	0.00	0.08	0.00
Living Situation	0.06	0.11	0.26
Medical	0.10	**0.52**	0.11
Motor	0.03	0.24	0.11
Organizational Complexity	0.01	0.01	**0.31**
Physical	0.03	0.05	**0.31**
Recreational Play	0.01	0.15	0.11
Regulatory	0.03	0.28	0.28
Sensory Reactivity	0.00	0.09	0.06
Sleep	0.08	0.16	0.21
Social Functioning	0.03	0.14	**0.30**
Treatment Involvement	0.01	0.28	0.01
**Risk Behaviors**			
Aggressive Behavior	0.03	0.07	0.20
Birth Weight	0.06	0.22	0.08
Intentional Misbehavior	0.01	0.04	0.17
Labor and Delivery	0.06	0.18	0.08
Length of Gestation	0.07	0.15	0.08
Maternal Availability	0.17	0.23	0.25
Parental Care	0.13	0.24	0.20
PICA	0.00	0.01	0.01
Self-Harm	0.00	0.01	0.02
Substance Exposure	0.22	0.24	0.25

*Note*: Within class, bolded values have a ≥ 0.3 probability of being actionable

^a^Actionable refers to scores of 2 or 3 in the Child and Adolescent Needs and Strengths assessment, meaning that immediate action needs to be taken because the child is at risk within that item

The “strengths” in the CANS were summarized by LCA class noting that resilience was a strength item, although all remaining items contribute to the larger concept of resilience (see Table 3). Actionability was defined as those with a probability of less than 0.7, meaning that above 0.7 the child demonstrated the strength factor. Within Class 1, “family nuclear strength” was actionable. There were no strengths which required attention or development detected in Class 2. Within Class 3, all the strengths were actionable except for “curiosity” and “resiliency” which were 0.79 and 0.74, respectively.

**Table 3:** Probabilities of useful strengths by class membership of children five years and younger from the Child and Adolescent Needs and Strengths assessment in a large midwestern state from 2011–2021

	Class 1	Class 2	Class 3
	n = 2531 (58%)	n = 503 (12%)	n = 1306 (30%)
	Resilient	Unfolding	Missed
Strengths			
Adaptability	0.95	0.78	**0.58**
Curiosity	0.99	0.88	0.79
Family Extended Strength	0.74	0.76	**0.63**
Family Nuclear Strength	**0.67**	0.74	**0.68**
Interpersonal	0.97	0.83	**0.63**
Persistence	0.96	0.83	**0.66**
Relationships Permanence	0.72	0.75	**0.66**
Resiliency	0.98	0.89	0.74

*Note*: Within class, bolded values have a ≤ 0.7 probability of being actionable

^a^Actionable refers to scores of 2 or 3 in the Child and Adolescent Needs and Strengths assessment, meaning that the child needs to build strengths and action is needed

**Fig. 3:**
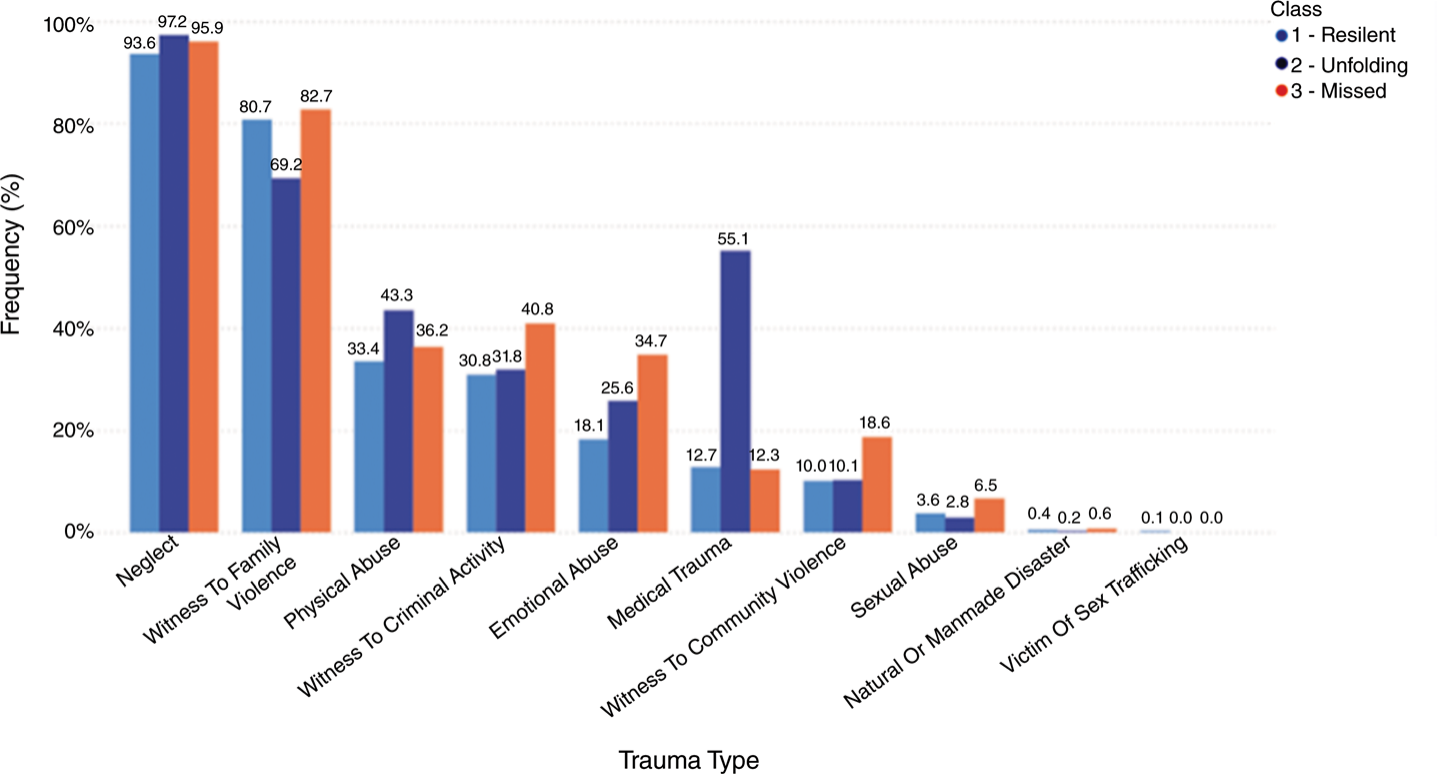
Frequency of trauma type per LCA class (‘resilient’, ‘unfolding’, ‘missed’) in children less than five-years of age: the Child and Adolescent Needs and Strengths (CANS) comprehensive assessment from a large mid-western state in the United States (2011–2021)

We evaluated frequency and type of trauma experienced by Class determined in the LCA (See Fig. 3). “Neglect” and “witness to family violence” were documented across all classes. Class 2 demonstrated the highest frequency of “medical traumas” and “physical abuse”. The traumas of “witness to criminal activity” and “emotional abuse” were more frequently documented in children who were in Class 3 than other classes. The remaining traumas were distributed across all classes equally.

Class 1 comprised the largest proportion of the sample and was labelled a *resilient* group of children because of the high percentage of strengths and lowest actionable number of needs. This class also had the lowest proportion of males compared to the other classes. Class 2 was characterized by children with developmental challenges and the highest frequency of medical traumas. Children in this class also had marginally higher rates of physical abuse and neglect. We propose that this group may be developing traumatic stress, but because of the nature of trauma and the presence of developmental delays, it was harder to detect at the time of assessment. For this reason, Class 2 was labelled as “*unfolding*”. Class 3 was a group of children with behavioral health needs that often mirrored or overlapped with traumatic stress symptoms; however, these children were not identified as having symptoms despite documented CIT exposure. Class 3 was labelled as “*missed*”. This class also was characterized by higher levels of violence in their traumatic experiences (e.g., witness to family violence, community violence and criminal activity). This group was the oldest (3.1 years) and had the highest proportion of white children (58%). In addition to having developmental functioning challenges as Class 2, the detection of “attachment” as a behavioral and emotional need was highlighted in this group. This factor and need in “communication” functioning item distinguish the class from the unfolding group (Class 2). In addition, children in Class 3 had the most actionable strengths, observed as strengths needing to be developed, compared to the other two classes.

## Discussion

Using a population-based sample, this study found an association between traumatic exposures (captured using CANS items that correspond to ACEs) and detection of traumatic stress of very young children taken into state custody. The power and ability for the CANS to predict health outcomes for children with CIT are well established [39–41, 42–45]. To optimize the ability to provide person-centered, trauma-informed care, our study’s primary objective was to identify patterns of functional and asset-based factors in children housed in foster care who have documented CIT exposure but do not demonstrate subsequent symptoms of traumatic stress. We characterized three groups of children under five years of age based on these functional and asset-based factors. The characteristics of each class have important implications for the design of child welfare systems.

Class 1, as the ‘resilient group’, demonstrated only one actionable need, which was “family nuclear life functioning”. Related to this need, the only actionable strength (i.e., strength needing development) was “family nuclear strength”. These findings combined with having the higher proportion of females are consistent with a child welfare sample, such that, other than family challenges, these young children are doing well [29, 56]. The results of this study identified that a notable proportion of children with CIT history are quite resilient in the face of adversity: they have few behavioral/mental health needs and demonstrate several strengths which contribute to asset-based resilience. We suggest these children will be best served through strategies which emphasize maintenance of strengths and monitoring of family functioning. Of course, the present study is cross-sectional; therefore, we know little about the long-term resilience of these children. Longitudinal research is needed to see whether these resilient children remain resilient or ultimately experience traumatic stress later in life as previously identified [10].

Children with cognitive, physical, and emotional disabilities have been shown to have higher frequencies of ACEs than those without special needs [56]. In addition, children with disabilities are over three times more likely to experience maltreatment, and they are disproportionately represented in child welfare services [46]. Children with developmental delays may communicate symptoms of traumatic stress differently than those without neurodevelopmental anomalies. Class 2 children were also more likely to have medical needs which was a distinct factor in this class. Further, over 36% of this class were identified as by Black/African American males, which is higher than reported in investigations of older children [47]. Further research is necessary to explore this disparity, as racial bias has been described in the child welfare system [48–50]. It is notable that this group represents both children with minority and disability status both of which have been shown to be experience bias in clinical settings [48, 50]. These children demonstrated assets across all strength items upon entry to child welfare services. Although in Class 2 the level of strengths was lower than Class 1, it is possible that these children are resilient children with developmental and medical challenges. However, we proposed that differentiation of traumatic stress symptoms may be secondary to identification of developmental delays or medical symptoms and may be complicated by implicit bias. In other words, the developmental needs of the children may be creating ‘noise’ which may obscure the detection of symptoms of traumatic stress [50]. Further, child welfare professionals also may be less cognizant of traumatic stress symptoms associated with medical traumas. Another difference between this class and the third class is that strengths are often present [51, 52].

Class 3 children had the fewest strengths. We also saw 30% higher emotional abuse and 57% higher sexual abuse traumatic exposure in this sample. Attachment behavior and oppositional behaviors related to family extended life are the most common mental health presentations of young children in the missed class who experienced CIT. These common behavioral symptoms likely provide the greatest challenge to detecting traumatic stress in young children; however, the treatment of traumatic stress can be quite different from traditional mental healthcare [53]. Class 3 was the only group with notable needs on the organizational functioning item. This indicated that 31% of these children were engaged in care in multiple systems and there were problems with care coordination across these systems.

The LCA approach permitted us to characterize these children in terms of behavior, health status, exposure to traumatic events, and development. As an advantage, the LCA considered population heterogeneity across multiple factors rather than describing variability of a single variable. As a person-centered approach, the LCA characterized a population across a subset pattern underlying which there is an optimal number of latent classes to represent that population [31, 26, 54, 55]. Like prior work [29, 31], we were able to understand the co-occurrence of CIT more deeply in a nuanced manner in very young children. This person-centered approach permitted the identification of heterogenous sub-groups of children who did not have detectable traumatic stress. Evidence of high need and actionable strength in the unfolding and missed classes in our study supported the cumulative risk hypothesis which is associated with poorer health and emotional outcomes, even when controlled for demographic factors and special medical care requirements [56]. Most children with undetected traumatic stress may simply be resilient, and therefore, demonstrate fewer symptoms. When trauma-affected children developed strengths in relationships, they exhibit greater adaptation to negative exposures (such as fewer behavioral needs, reductions in mental health challenges 33, 34). In this context, the results demonstrate the importance of assessing both traumatic stress and strengths to ensure that children who are resilient are not referred into the same interventions as those who are struggling with their adjustment to adverse life events.

The identification of a large group of resilient children bolsters the case for creating stability in an inherently unstable system where what is best for the children often is to remove them from homes and provide residential care. By focusing on asset-based factor development to mitigate traumatic stress symptoms, this study suggests changing the paradigm of person-centered care to asset-based rather than a deficit-based approach which focuses primarily on need associated with diagnosed conditions [39–41]. Asset-based resilience is likely a factor of moderators including family functioning and interpersonal relationships which are commonly impaired with CIT exposure [10]. Furthermore, consideration of age and pervasiveness of effects of traumatic exposures [31] highlights the continued need to derive effective and efficacious interventions which are trauma-informed for children in foster care systems. Child welfare professionals may require training and support to help them work effectively with very young children in foster care. This additional support may be especially important in helping professionals to distinguish between behavioral/emotional challenges and traumatic stress symptoms. Considering the balance among social, cultural, and other identity factors, in the context of the detection of behavioral and emotional challenges, should remain a priority [57]. These findings suggest that these profiles are critical in identifying when very young children are likely to have traumatic stress and should be carefully considered when implementing treatment plans.

## Limitations

The current study has several limitations which should be addressed with additional research. It is a cross-sectional approach to the challenge of traumas in very young children and cannot establish causality among factors. However, our intention was not to establish causality, rather to understand the child welfare systems’ ability to detect traumatic stress symptoms in very young children. Future research should follow these children over time to see whether the resilient children stay resilient to these traumatic experiences and whether, when, and how traumatic stress manifests in the group we proposed as ‘unfolding’. Similar research would help to validate that traumatic stress was indeed not identified in the group we proposed as ‘missed’.

Although the classification certainty of our optimal model was within standards, there always exists a level of uncertainty for class separation. When we reviewed the three and four class solutions, we chose the three class LCA because we were able to describe three distinct and interpretable patterns. As such, we believe that our findings are robust and supported. Other researchers with different data sets may find different patterns and number of class solutions as seen previously [27–30]. Children in this age group are represented by a caregiver or guardian and may not accurately reflect the child’s experience with trauma. We used the trauma/ACEs domain in the CANS. Although the definitions are the same, the assessment processes can be different than other ACEs measures. Our findings are like others which are nationally and state-based samples in describing traumatic stress and trauma exposures. Future studies should consider if different patterns arise when considering indicators which vary from this study’s approach.

Child welfare systems vary, and these findings may not generalize to other areas in the United States. These data had representation of fewer ethnic minorities compared to some regions of the US. Prior LCA analyses adjusted for race and ethnicity demonstrated high need and low strengths using the CANS in children in the welfare system [28, 31, 55]. Although our data were not adjusted for race, we propose that the findings remain. Age can also impact the expression of traumatic stress symptoms [58]. The present study was an analysis of data collected on the needs and strengths of children as they entered child welfare settings. It is likely entry-point assessment may not capture the full breadth and depth of a child’s adverse experiences and traumatic stress symptoms. This challenge might be particularly acute for children under five years old, especially those who may not have a reliable historian available and when the child may not verbalize or otherwise outwardly demonstrate assessable characteristics.

## Conclusions

Understanding and addressing traumatic stress in children is an important public health priority. The present study demonstrates how one state has built the capacity to assess and detect traumatic stress for children served in the child welfare system. Given the widespread use of the CANS in child welfare, this capacity exists in most states and internationally. Understanding the challenges of detecting traumatic stress in very young children will help us provide improved decision support, training, and supervisory support to caseworkers and other system partners to develop their detection skills. Further understanding how children become resilient in the face of adversity, even at a very young age, should help us to clarify how to help all children achieve and maintain factors of resiliency through intentional and directed trauma-informed interventions.

## Data Availability

Data is available within the article. A confidentiality agreement (i.e., “Data Use Agreement”) restricts public sharing of full data sets generated and/or analyzed during the current study.

## References

[1] McKay MT, Cannon M, Chambers D, Conroy RM, Coughlan H, Dodd P (2021). Childhood trauma and adult mental disorder: a systematic review and meta-analysis of longitudinal cohort studies. Acta Psychiatrica Scandinavica.

[2] Larson S, Chapman S, Spetz J, Brindis CD (2017). Chronic Childhood Trauma, Mental Health, Academic Achievement, and School-Based Health Center Mental Health Services. J Sch Health.

[3] Doom JR, Cicchetti D. The developmental psychopathology of stress exposure in childhood. In: Harkness K, Hayden EP, editors. The Oxford Handbook of Stress and Mental Health. Oxford University Press; 2018.

[4] Copeland WE, Keeler G, Angold A, Costello EJ (2007). Traumatic events and posttraumatic stress in childhood. Arch Gen Psychiatry.

[5] Felitti VJ, Anda RF, Nordenberg D, Williamson DF, Spitz AM, Edwards V (1998). Relationship of childhood abuse and Household Dysfunction to many of the leading causes of death in adults: the adverse childhood experiences (ACE) study. Am J Prev Med.

[6] Meinck F, Cosma AP, Mikton C, Baban A (2017). Psychometric properties of the adverse childhood experiences abuse short form (ACE-ASF) among Romanian high school students. Child Abuse Negl.

[7] Morris AS, Williamson AC (2018). Building early social and emotional relationships with infants and toddlers.

[8] Pace CS, Muzi S, Rogier G, Meinero LL, Marcenaro S (2022). The adverse childhood experiences – International Questionnaire (ACE-IQ) in community samples around the world: a systematic review (part I). Child Abuse Negl.

[9] SAMHSA. Understanding Child Trauma 2022 [cited 2022 January 31]. Available from: https://www.samhsa.gov/child-trauma/understanding-child-trauma.

[10] Cook A, Spinazzola J, Ford J, Lanktree C, Blaustein M, Cloitre M (2005). Complex trauma in children and adolescents. Psychiatric Annals.

[11] The National Child Traumatic Stress Network. Complex Trauma Effects 2021 [Available from: https://www.nctsn.org/what-is-child-trauma/trauma-types/complex-trauma/effects.

[12] American Pyschological Association. Trauma 2022 [Available from: https://www.apa.org/topics/trauma.

[13] Lubit R, Rovine D, Defrancisci L, Eth S (2003). Impact of trauma on children. J Psychiatr Pract.

[14] Bartlett JD, Griffin JL, Spinazzola J, Fraser JG, Noroña CR, Bodian R (2018). The impact of a statewide trauma-informed care initiative in child welfare on the well-being of children and youth with complex trauma. Child Youth Serv Rev.

[15] Washington Fosters. Understanding trauma in foster children2020 January 26, 2022. Available from: https://foster.wachildrenandfamilies.org/blog/understanding-trauma-in-foster-children.

[16] Greeson JKP, Briggs EC, Kisiel CL, Layne CM, Ake Iii GS, Ko SJ (2011). Complex Trauma and Mental Health in children and adolescents placed in Foster Care: findings from the National Child Traumatic Stress Network. Child Welfare.

[17] Takarangi M, Strange D, Lindsay DS (2014). Self-report may underestimate trauma intrusions. Conscious Cogn.

[18] Us Department of Health and Human Services. Healthy People 2030: Social and Community Context [Available from: https://health.gov/healthypeople/objectives-and-data/browse-objectives/social-and-community-context.

[19] Joseph S, Knibbs J, Trauma JH, Hosin AA (2007). Resilience and growth in children and adolescents. Responses to traumatized children.

[20] Leon SC, Ragsdale B, Miller SA, Spacarelli S (2008). Trauma resilience among youth in substitute care demonstrating sexual behavior problems. Child Abuse Negl.

[21] Lou Y, Taylor EP, Di Folco S (2018). Resilience and resilience factors in children in residential care: a systematic review. Child Youth Serv Rev.

[22] Morris AS, Hays-Grudo J, Zapata MI, Treat A, Kerr KL (2021). Adverse and protective childhood experiences and parenting attitudes: the role of cumulative protection in understanding resilience. Advers Resil Sci.

[23] Blaustein ME, Kinniburgh KM (2019). Treating traumatic stress in children and adolescents: how to foster resilience through attachment, self-regulation, and competency.

[24] Hudek N. Risk and resilience in the internalizing outcomes of children in out-of-Home Care. Université d’Ottawa/University of Ottawa; 2018.

[25] Allen JA, Augustin T. So much more than cheap labor! Volunteers engage in emotional labor. Social Sci J. 2021:1–17.

[26] Nylund KL, Asparouhov T, Muthén BO (2007). Deciding on the number of classes in latent class analysis and growth mixture modeling: a Monte Carlo Simulation Study. Struct Equ Model.

[27] Hong S, Walton B, Kim H-W, Lee S, Rhee TG. Longitudinal patterns of strengths among Youth with Psychiatric disorders: a latent Profile Transition Analysis. J Child Psychiatry Hum Dev. 2021:1–8. 10.1007/s10578-021-01217-3 34255230

[28] Shimshock S, Chor KHB, Brylske PD (2022). Using latent class analysis to identify clinical subgroups and pathways of youth in a therapeutic foster care program. Child Youth Serv Rev.

[29] Nguyen T, Basson P, Perry D. D. Patterns of Trauma Among Youth Seeking Mental Health Services at a Community-Based Clinic: A Latent Class Analysis Approach. Research on child and adolescent psychopathology. 2022. 10.1007/s10802-022-00998-y 36401776

[30] Lanier P, Maguire-Jack K, Lombardi B, Frey J, Rose RA (2018). Adverse childhood experiences and Child Health outcomes: comparing cumulative risk and latent class approaches. Matern Child Health J.

[31] Gordon CT, Nguyen PT, Mitchell AK, Tyler PM. Profiles of childhood adversity and associated psychopathology in youth entering residential care. Psychol Trauma. 2022. 10.1037/tra0001325 35901426

[32] Lyons JS, Communimetrics. A Communication Theory of Measurement in Human Service Settings. 1. Aufl. ed. New York, NY: New York, NY: Springer-Verlag; 2009.

[33] Lam A, Lyons JS, Griffin G, Kisiel C (2015). Multiple traumatic experiences and the expression of traumatic stress symptoms for children and adolescents. Residential Treat Child Youth.

[34] Kisiel C, Patterson N, Torgersen E, den Dunnen W, Villa C, Fehrenbach T (2018). Assessment of the complex effects of trauma across child serving settings: measurement properties of the CANS-Trauma Comprehensive. Child Youth Serv Rev.

[35] Alamdari G, Kelber MS (2016). The child and adolescent needs and strengths as an outcome measure in community mental health: factor analysis and a validation of the short form. Commun Ment Health J.

[36] Kisiel C, Fehrenbach T, Small L, Lyons JS (2009). Assessment of complex trauma exposure, responses, and service needs among children and adolescents in child welfare. J Child Adolesc Trauma.

[37] Lyons JS. Transformational Collaborative Outcomes Management. Transformational collaborative outcomes management: managing the business of personal change. Springer; 2022. pp. 59–95.

[38] Lyons JS (2009). A communication theory of measurement in human service settings.

[39] Ellis BH, Fogler J, Hansen S, Forbes P, Navalta CP, Saxe G (2012). Trauma systems therapy: 15-month outcomes and the importance of effecting environmental change. Psychol Trauma.

[40] Troy JD, Torrie RM, Warner DN (2021). A machine learning approach for identifying predictors of success in a Medicaid-funded, community-based behavioral health program using the child and adolescent needs and strengths (CANS). Child Youth Serv Rev.

[41] McIntosh A, Lyons JS, Weiner DA, Jordan N (2010). Development of a Model for Predicting running away from residential treatment among children and adolescents. Residential Treat Child Youth.

[42] Anderson RL, Estle G (2001). Predicting Level of Mental Health Care among Children served in a delivery system in a rural state. J Rural Health.

[43] Burnett-Zeigler I, Lyons JS (2010). Caregiver factors Predicting Service utilization among Youth participating in a School-based Mental Health Intervention. J Child Fam stud.

[44] Chor KHB, McClelland GM, Weiner DA, Jordan N, Lyons JS (2012). Predicting outcomes of children in residential treatment: a comparison of a decision support algorithm and a multidisciplinary team decision model. Child Youth Serv Rev.

[45] Toche-Manley LL, Dietzen L, Nankin J, Beigel A (2014). Are two voices Better Than one? Predicting Permanency in Minority Youth Using Multi-informant Mental Health and Strength Data. J Behav Health Serv Res.

[46] Berg KL, Shiu CS, Msall ME, Acharya K (2015). Victimization and depression among youth with disabilities in the US child welfare system. Child: Care Health & Development.

[47] Berg KL, Medrano J, Acharya K, Lynch A, Msall ME (2018). Health impact of participation for vulnerable youth with disabilities. Am J Occup Therapy.

[48] Berger Lawrence M, McDaniel M, Paxson C (2005). Assessing parenting behaviors across racial groups: implications for the child Welfare System. Social Service Review.

[49] Bartholet E, Wulczyn F, Barth RP, Lederman CJCHIB. June. Race and child welfare. 2011.

[50] Rosenthal CM, Parker DM, Thompson LA (2022). Racial disparities in Child Abuse medicine. JAMA Pediatr.

[51] Go M, Chu CM, Barlas J, Chng GS (2017). The role of strengths in anger and conduct problems in maltreated adolescents. Child Abuse Negl.

[52] Dorsey S, Burns BJ, Southerland DG, Cox JR, Wagner HR, Farmer EM (2012). Prior trauma exposure for Youth in Treatment Foster Care. J Child Fam stud.

[53] Griffin G, McClelland G, Holzberg M, Stolbach B, Maj N, Kisiel C (2011). Addressing the impact of trauma before diagnosing mental Illness in child welfare. Child Welfare.

[54] Lanza ST, Cooper BR (2016). Latent class analysis for Developmental Research. Child Dev Perspect.

[55] Katz CC, Lalayants M, Lushin V (2021). The longitudinal effects of maltreatment class membership on post-traumatic stress & depression. Child Abuse Negl.

[56] Balistreri KS, Alvira-Hammond M (2015). Adverse childhood experiences, family functioning and adolescent health and emotional well-being. Public Health (London).

[57] Choi KR, Graham-Bermann SA (2018). Developmental considerations for assessment of trauma symptoms in preschoolers: a review of measures and diagnoses. J Child Fam stud.

[58] Huguenel BM, Leon SC, Hindt LA, Lutz N, Osborne J (2021). Profiles of Maltreatment in the child Welfare System: Predicting Mental Health outcomes and examining age as a moderator. J Trauma Stress.

